# ERCC1 rs11615 polymorphism and chemosensitivity to platinum drugs in patients with ovarian cancer: a systematic review and meta-analysis

**DOI:** 10.1186/s13048-021-00831-y

**Published:** 2021-06-21

**Authors:** Yuqiang Zhang, Sufen Cao, Chunyu Zhuang, Jiacheng Chen, Xiaojing Chen, Hong Sun, Shengying Lin, Bailang Lin

**Affiliations:** 1 Department of Obstetrics, Haikou Maternal and Child Health Hospital, Haikou, Hainan China; 2 Department of Nursing, Haikou Maternal and Child Health Hospital, No. 6 Wentan Road, Haikou, 570203 Hainan China; 3 Department of Hepatological Surgery, Hainan Provincial People’s Hospital, Haikou, Hainan China; 4 Department of Gynecology and Obstetrics, Haikou Maternal and Child Health Hospital, Haikou, Hainan China; 5 Department of Operation, Haikou Maternal and Child Health Hospital, Haikou, Hainan China

**Keywords:** Ovarian cancer, ERCC1, Platinum drugs, Meta-analysis

## Abstract

**Objective:**

To explore the relationship between ERCC1 rs11615 polymorphism and chemosensitivity to platinum drugs in ovarian cancer by the method of meta-analysis.

**Methods:**

Pubmed, Web of Science, EMBASE, Cochrane Library, China National Knowledge Infrastructure (CNKI), and China Wanfang databases were comprehensively searched up to September 2020, to identify the relationship between ERCC1 rs11615 polymorphism and chemosensitivity of ovarian cancer. The data was analyzed by Stata 15.0 statistic software.

**Results:**

A total of 10 published papers were included, including 1866 patients with ovarian cancer. The results showed that compared allele C at ERCC1 rs11615 locus with allele T, the pooled OR was 0.92 (95%CI:0.68 ~ 1.24, *P* > 0.05). There were no significant differences in recessive, dominant, homozygous, and heterozygous models. In accordance with a subgroup analysis of Ethnicity, all genotypes were statistically significant in the Asian population. In the allelic, dominant, recessive, homozygous and heterozygous models, the OR was 0.70 (95%CI:0.51 ~ 0.95), 0.20 (95%CI:0.07 ~ 0.56), 0.79 (95%CI:0.63 ~ 1.00), 0.21 (95%CI:0.07 ~ 0.59), 0.19 (95%CI:0.07 ~ 0.54), respectively, while in the Caucasian population, no statistically significant genotype was found.

**Conclusion:**

The ERCC1 rs11615 polymorphism is associated with chemosensitivity in patients with ovarian cancer, especially in the Asian population, but not in the Caucasian population.

## Introduction

The ovarian malignant tumor is one of the common malignant tumors of female reproductive organs, which is a severe threat to women’s health. Its incidence is second only to cervical cancer and uterine cancer, and its mortality ranks first among gynecological malignant tumors [1]. According to the latest data, there are about 300,000 cases of female ovarian cancer each year worldwide, with a death rate of 185,000 and a mortality rate of 0.45% [1]. Due to ovarian cancer’s insidious onset and lack of early diagnostic indicators, most of them are diagnosed in the late stage. The pathogenic mechanism of ovarian cancer is obscure, and it is generally believed to be the result of heredity and environment [2, 3]. At present, platinum-based combination chemotherapy is the first-line treatment for ovarian cancer after cytoreductive surgery. However, in the initial treatment period, 20% of patients do not respond to platinum chemotherapy, and up to 75% of patients with ovarian cancers have a relapse. Meanwhile, patients with the treatment after the relapse tend to have resistance to platinum drugs [4, 5]. If the drug resistance of ovarian cancer patients can be predicted before or at the initial stage of chemotherapy in order to adjust the chemotherapy scheme in time, the tumor remission rate can be enhanced, and the prognosis of patients can be improved [6, 7]. Therefore, identifying the sensitivity of patients to platinum drugs is the key to effectively enhance the efficacy of chemotherapy for ovarian cancer. Platinum drugs have been the most widely used chemotherapeutic drugs since they were put into clinical application in the 1970s [8, 9]. Clinically, platinum drugs have been extensively used in the treatment of malignant tumors such as nasopharyngeal carcinoma [10], esophageal cancer [11], breast cancer [12], etc. Therefore, platinum drugs have become a symbol of an era among anticancer drugs [13]. Platinum compounds (carboplatin or cisplatin) exert their antitumor effect through combining with DNA to form intrachain and interchain crosslinking and Pt–DNA adducts [14]. DNA adducts cause structural changes in DNA, which affects DNA replication and inhibits DNA synthesis. There are many factors in the development of tumor cells to platinum resistance, which may include a decrease of drug accumulation, an increase of glutathione level and metallothionein, and an improvement of DNA repair ability [15]. However, it is believed that DNA repair is the fundamental contributor to clinical drug resistance in platinum-based therapy, and the difference in individual DNA repair ability is closely related to platinum prognosis in patients with ovarian cancer [5]. Moreover, studies have confirmed that the clinical remission rate of tumor patients is related to the level of Pt–DNA adducts in their blood circulation [16]. If the DNA repair ability of tumor cells is reduced, the clearance of Pt–DNA adducts in blood circulation will be diminished, which leads to the augmented curative effect of platinum chemotherapeutic drugs. Otherwise, the curative effect will be unsatisfactory [16, 17]. Therefore, the DNA repair ability is the main factor that affects the efficacy of platinum drugs.

Nucleotide excision repair (NER) is one of the classic ways to participate in multi-drug resistance in ovarian cancer [18]. As an essential member of this pathway, ERCC1 gene expression products exert such function to recognize DNA damage in the initial repair stage [19]. Platinum-based chemotherapeutics take the role of an anti-cancer way mainly by cross-linking cancer cell DNA [18]. Previous studies have shown that changes in the third base (19007C/T) of the fourth exon of the ERCC1 gene can reduce its gene transcription activity by 50%, leading to a decrease in its protein or mRNA expression and affect chemosensitivity [20]. Therefore, the 19007C/T polymorphism of the ERCC1 gene is theoretically related to the sensitivity of platinum-based combined chemotherapy. However, the conclusions of previous studies on the ERCC1 rs11615 polymorphism are not consistent. Steffensen et al. [21] showed that the chemotherapeutic responses of the ovarian cancer patients with genotype TT at ERCC1 rs11615 locus were better than those with genotype TC + CC, while Kang et al. [22] believed that ovarian cancer patients with genotype CT + TT were more sensitive to platinum chemotherapy than those with genotype CC. Therefore, we systematically reviewed the related literature in this study, applying meta-analysis to conduct a comprehensive analysis upon the relationship between the ERCC1 rs11615 polymorphism and the chemosensitivity of platinum drugs in patients with ovarian cancer.

## Methods

### Literature retrieval

Pubmed, Web of Science, EMBASE, Cochrane Library, China National Knowledge Infrastructure (CNKI), and China Wanfang databases were searched for literature about ERCC1 rs11615 polymorphism and chemosensitivity of ovarian cancer, from inception to September 2020. The search strategy was as follows: (“ERCC1” OR “excision repair cross-complementation group 1”) AND (“ovarian carcinoma” OR “ovarian cancer” OR “ovarian tumor”) AND (“polymorphism” OR “gene mutation”. There was no language limitation. Two researchers independently searched and cross-checked the literature. When the two researchers disagreed with each other, they resolved disagreements through discussion, or turned to a third researcher for verification.

### Inclusion and exclusion criteria

#### Inclusion criteria

(1) patients with ovarian cancer confirmed by pathological examination; (2) patients received platinum-based combined chemotherapy after operation; (3) genotypes were detected by genomic DNA extracted from tumor tissue or peripheral blood; (4) studies with a large number of cases were selected if the same samples were used in multiple articles; (5) the target gene polymorphism was ERCC1 rs11615 locus or 19007C/T.

#### Exclusion criteria

(1) reviews, letters and conference summaries, etc.; (2) repeated reports, insufficient data, too little reported information, and unavailable literature; (3) studies with Newcastle–Ottawa scale (NOS) scores less than 6.

### Literature quality evaluation

The quality of the included studies was evaluated according to NOS [23], which was carried out independently by two researchers, and finally cross-checked. When there was a disagreement, it was resolved through discussion, or it was decided by a third researcher.

### Data extraction

All literature data were extracted independently by two researchers and finally cross-checked. The following information was extracted from each included literature: author, year of publication, country of the first author, patient ethnicity, total number of cases, chemotherapy regimen, genotype detection methods, and the number of patients with genotype sensitivity and drug resistance.

### Statistical analysis

The data were analyzed by Stata 15.0 statistical software. The odds ratio (OR) and its 95%CI were calculated as effect size. Q-test was used to assess the heterogeneity across the studies included. If I^2^ ≥ 50%, or *P* ≤ 0.05, the heterogeneity was considered to exist, and thus a random-effects model (REM) was adopted. If I^2^ < 50% and *P* > 0.05, the heterogeneity was considered to be not significant, so a fixed-effects model (FEM) was used for data merging. If there was heterogeneity, subgroup analysis was carried out according to Ethnicity and Hardy–Weinberg Equilibrium (HWE) to explore the source of heterogeneity. Z-test was used to test the significance of the pooled OR value. Funnel plot and Egger’s Test were used to evaluate publication bias. The funnel plot was made by filling and trimming methods. Finally, a sensitivity analysis was conducted to verify the robustness of the research results.

## Results

### Features of the included studies

**Fig. 1:**
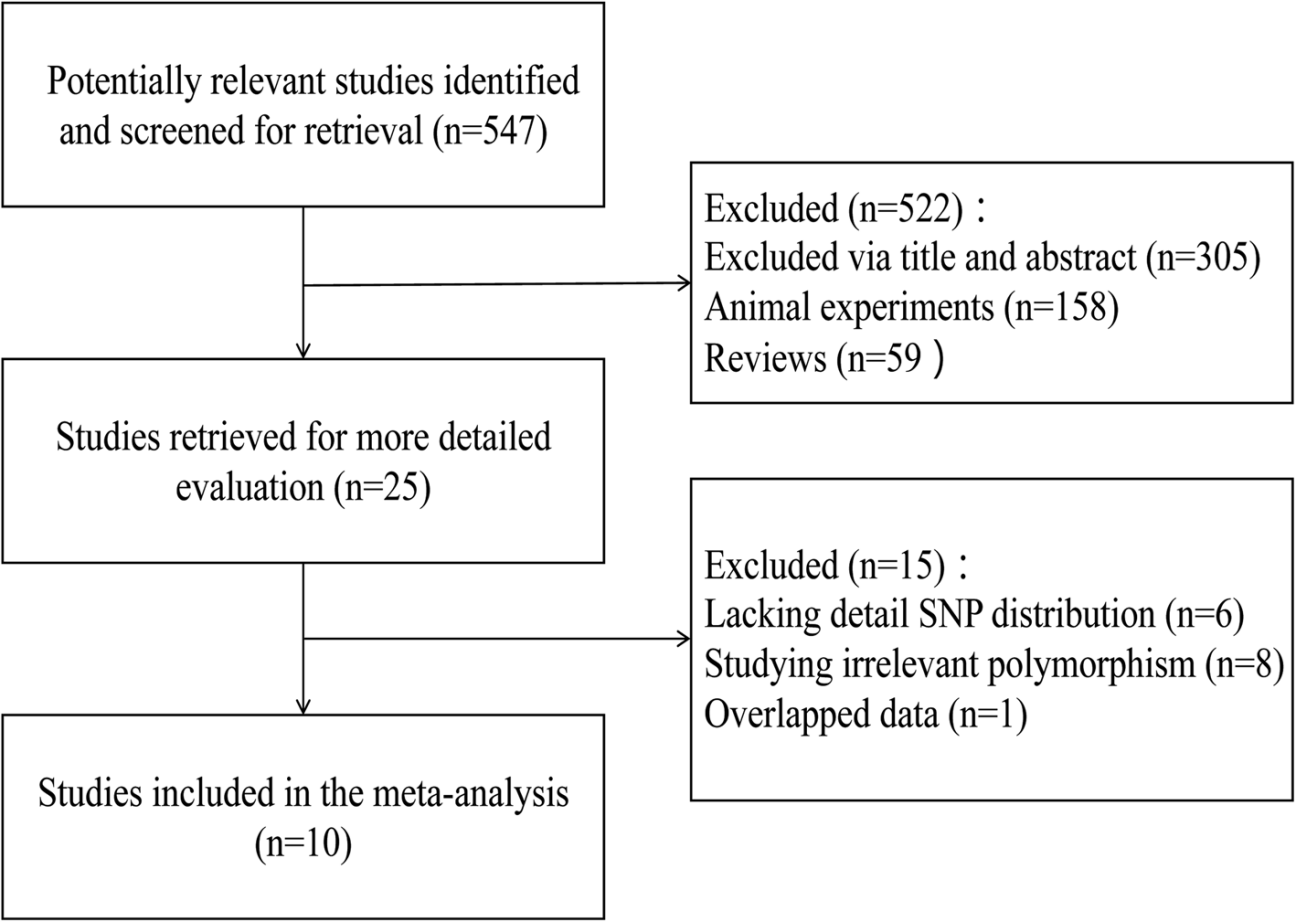
A flow diagram of the study selection process

According to the strict inclusion criteria, a total of 10 articles were included [21, 22, 24–31], including a total of 1866 patients with ovarian cancer. The specific literature screening process is shown in Fig. 1, and the basic characteristics of the included studies are shown in Table 1, from which it can be seen that the NOS scores of the included studies are all above 6.

**Table 1:** The basic characteristics of the inclusion studies

First Author	Year	Country	Tumor stage	Regimen of chemotherapy	Genotyping method	Nunber of cases	Resistant			Responder			P (HWE)	NOS score
							CC	CT	TT	CC	CT	TT		
Kang	2006	Korean	I-IV	platinum-based chemotherapy	Snapshot	60	15	5	0	20	16	4	0.763	8
Smith	2007	USA	I-IV	platium ± paditaxel chemotherapy	PCR–RFLP	176	11	22	15	23	60	45	0.701	8
Steffensen	2008	Danish	II-IV	carboplatin and cyclophosphamide combined-chemotherapy	TaqMan assay-based Real time PCR	100	3	7	2	12	34	42	0.239	8
Steffensen	2011	Danish	I-IV	Combined with carboplatin and paclitaxel	PCR–RFLP	157	2	4	7	15	71	58	0.321	8
Bösmüller	2011	Germany	I-III	Standard carboplatin-taxane	PCR–RFLP	41	6	5	3	7	9	11	0.098	8
Moxley	2013	USA	IIIB-IV	Platium- Based Chemotherapy	PCR–RFLP	64	10	13	9	14	8	10	0.005	8
Qi BL	2013	China	I-IV	Platinum-based combination	Snapshot	220	38	26	9	78	67	2	0.003	7
Huo XY	2017	China	I-IV	TP (paclitaxel / docetaxel + cisplatin / carboplatin / oxaliplatin)	PCR–RFLP	280	43	29	16	104	82	6	0.049	7
Yang SY	2017	China	III-IV	Cisplatin / carboplatin, combined with cyclophosphamide / paclitaxel	PCR–RFLP	209	28	31	12	70	67	1	0.000	7
Bao Y	2020	China	II-IV	cisplatin based Chemotherapy	PCR–RFLP	559	209	124	47	115	55	9	0.473	8

*NOS* Newcastle–Ottawa Scale, *PCR–RFLP* Polymerase chain reaction-restriction fragment length polymorphism, *HWE* Hardy–Weinberg equilibrium

### The results of meta-analysis

#### Allele comparison

**Fig. 2:**
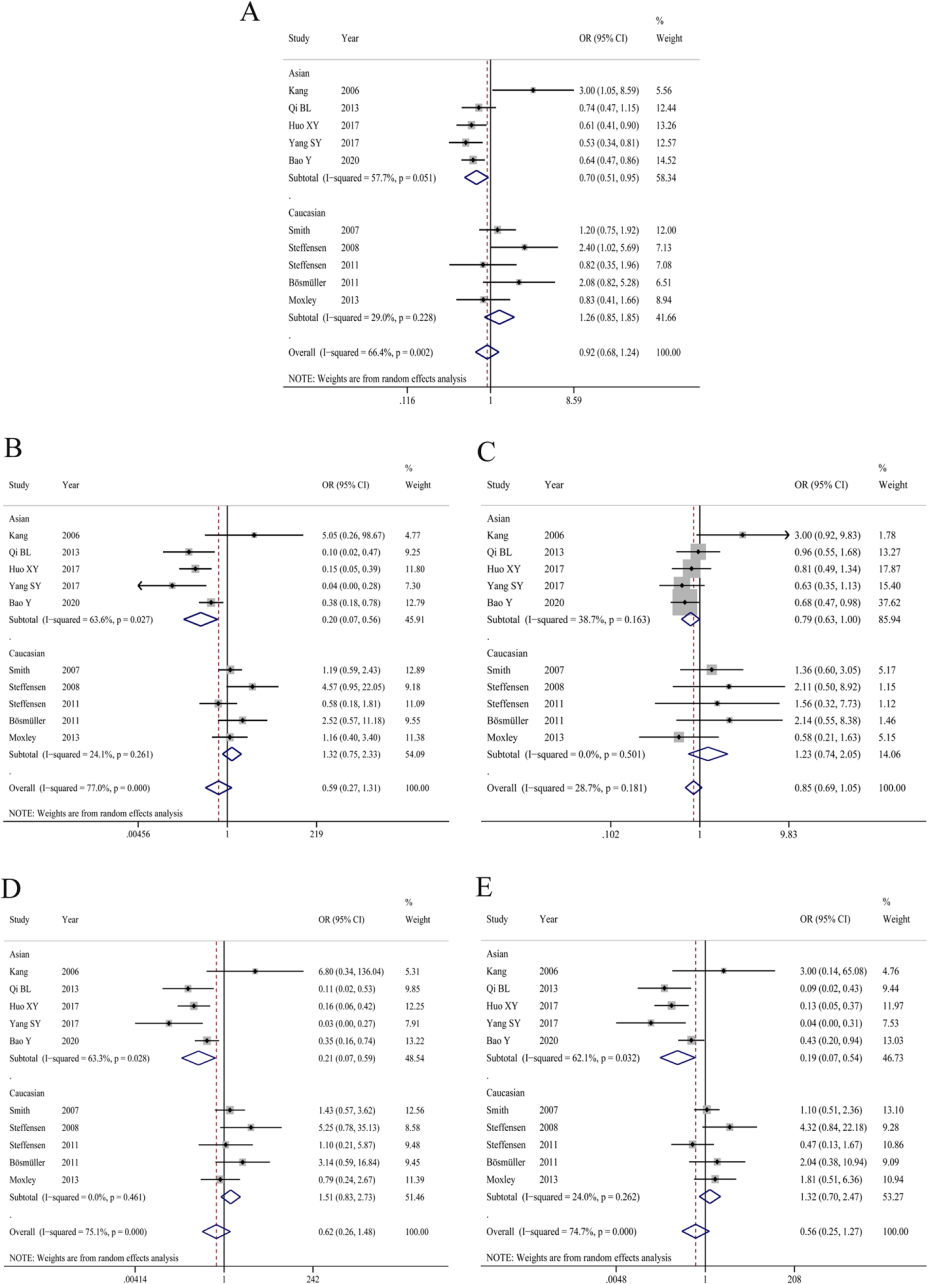
Forest plots for the association between ERCC1 Rs11615 polymorphism and chemosensitivity to platinum drugs in ovarian cancer (**A** allele model; **B** dominant gene model; **C** recessive gene model; **D** homozygous model; **E** heterozygous model)

**Fig. 3:**
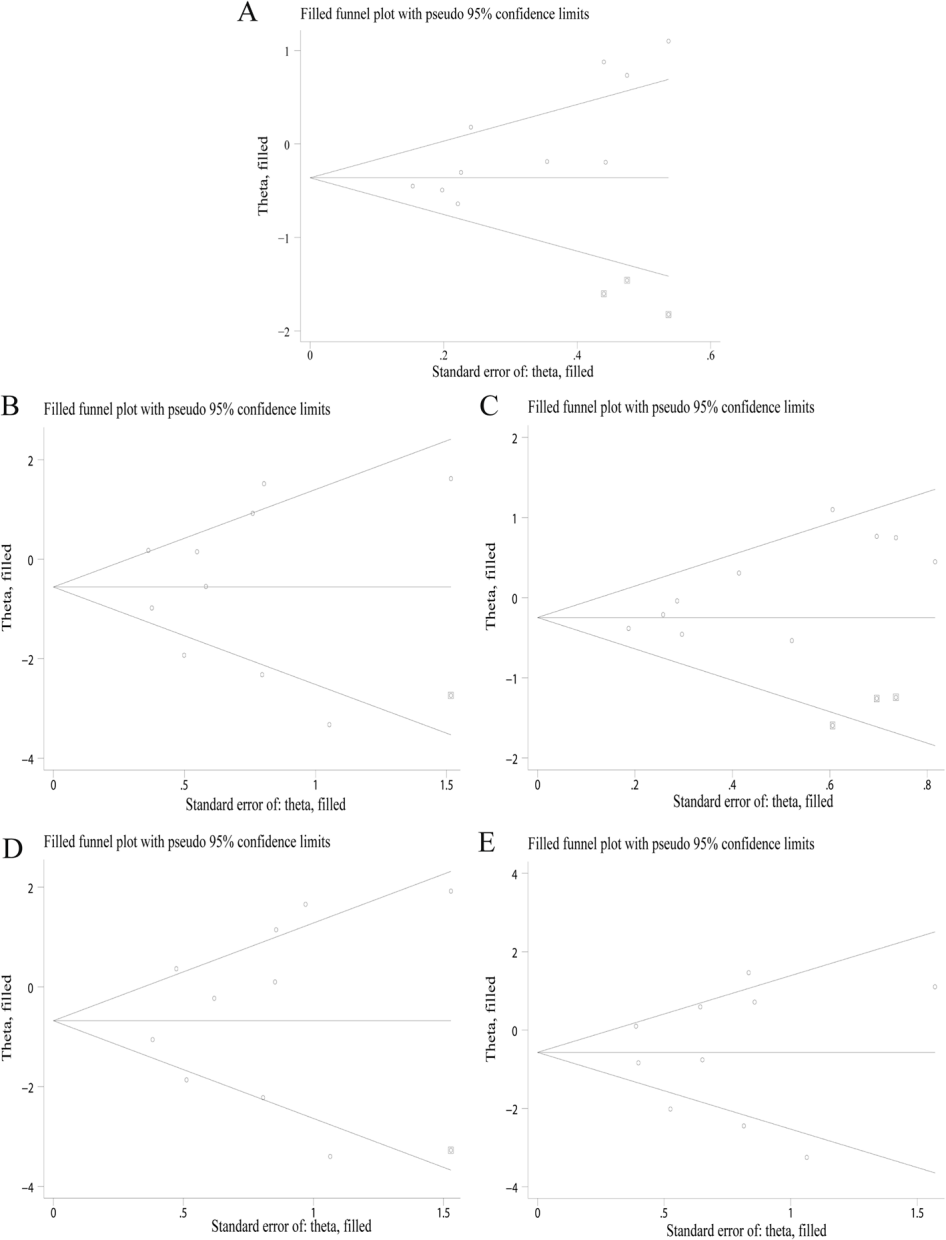
Funnel plots for the assessment of publication bias (**A** allele model; **B** dominant gene model; **C** recessive gene model; **D** homozygous model; **E** heterozygous model)

The main results of the meta-analysis were shown in Table 2 and Fig. 2. The heterogeneity test results showed that I^2^ = 66.4% (*P* < 0.05), which indicated significant differences in heterogeneities between studies, so the REM was adopted. The forest plot was shown in Fig. 2A. Compared with allele T, the chemosensitivity to platinum drugs of allele C of ERCC1 rs11615 was set as control. The results showed that OR = 0.92 (95%CI:0.68, 1.24, *P* > 0.05). Ethnic subgroup analysis showed that for the Asian population, OR = 0.70 (95%CI:0.51 ~ 0.95), the difference was statistically significant, and the heterogeneity also decreased. While in the Caucasian population, the difference was not statistically significant. Subgroup analysis of HWE showed that there was no notable decrease in heterogeneity. The symmetry of the funnel plot was general (Fig. 3A), and Egger’s Test showed that *P* < 0.05, which indicated that there was a certain publication bias.

**Table 2:** Meta-analysis of ERCC1 rs11615 polymorphism and chemosensitivity of ovarian cancer (subgroup analysis by ethnicity and HWE)

Genetic models	Subgroup	n	OR	95%CI	P_r_	I^2^ (%)	P_h_	Model	P_b_ (Egger’s Test)
C vs.T	Overall	10	0.92	0.68 ~ 1.24	0.574	66.4	0.002	REM	0.006
Ethnicity	Asian	5	0.70	0.51 ~ 0.95	**0.021**	57.7	0.051	REM	0.048
	Caucasian	5	1.26	0.85 ~ 1.85	0.246	29.0	0.228	FEM	0.463
HWE	Yes	7	1.12	0.73 ~ 1.73	0.601	73.9	0.001	REM	0.014
	No	3	0.65	0.49 ~ 0.87	0.003	0.0	0.433	FEM	0.557
CC + CT vs. TT	Overall	10	0.59	0.27 ~ 1.31	0.194	77.0	0.000	REM	0.165
Ethnicity	Asian	5	0.20	0.07 ~ 0.56	**0.002**	63.6	0.027	REM	0.315
	Caucasian	5	1.32	0.75 ~ 2.33	0.342	24.1	0.261	FEM	0.221
HWE	Yes	7	0.87	0.36 ~ 2.08	0.750	75.9	0.000	REM	0.060
	No	3	0.18	0.02 ~ 1.63	0.127	83.9	0.002	REM	0.260
CC vs. CT + TT	Overall	10	0.85	0.69 ~ 1.05	0.138	28.7	0.181	FEM	0.011
Ethnicity	Asian	5	0.79	0.63 ~ 1.00	**0.046**	38.7	0.163	FEM	0.052
	Caucasian	5	1.23	0.74 ~ 2.05	0.416	0.0	0.501	FEM	0.351
HWE	Yes	7	0.90	0.70 ~ 1.17	0.432	44.0	0.098	FEM	0.010
	No	3	0.75	0.52 ~ 1.10	0.139	0.0	0.522	FEM	0.674
CC vs. TT	Overall	10	0.62	0.26 ~ 1.48	0.281	75.1	0.000	REM	0.054
Ethnicity	Asian	5	0.21	0.07 ~ 0.59	**0.003**	63.3	0.028	REM	0.279
	Caucasian	5	1.51	0.83 ~ 2.73	0.175	0.0	0.461	FEM	0.230
HWE	Yes	7	1.04	0.39 ~ 2.82	0.935	75.1	0.000	REM	0.014
	No	3	0.16	0.02 ~ 1.08	0.059	76.2	0.015	REM	0.305
CT vs. TT	Overall	10	0.56	0.25 ~ 1.27	0.163	74.4	0.000	REM	0.269
Ethnicity	Asian	5	0.19	0.07 ~ 0.54	**0.002**	62.1	0.032	REM	0.436
	Caucasian	5	1.32	0.70 ~ 2.47	0.392	24.0	0.262	FEM	0.280
HWE	Yes	7	0.76	0.33 ~ 1.77	0.529	70.3	0.003	REM	0.147
	No	3	0.20	0.02 ~ 2.37	0.201	85.8	0.001	REM	0.350

*OR* Odds ratio, *HWE* Hardy–Weinberg equilibrium, *REM* Random-effects model, *FEM* Fixed-effects model, *P*_*r*_ P for OR, *P*_*h*_ P for Heterogeneity, *P*_*b*_ P for Publication bias

#### Dominant genetic model

In the dominant genetic model (CC + CT vs. TT)), genotype CC + CT was used as the exposure factor, genotype TT as the non-exposure factor, and chemosensitivity as the control. Heterogeneity test showed that I^2^ = 77.0% (*P* < 0.05), indicating the existence of the heterogeneity. Hence, a FEM model was adopted. The forest plot was as shown in Fig. 2B. The results showed that OR = 0.59 (0.27, 1.31, *P* > 0.05). The ethnic subgroup analysis showed that for the Asian population, OR = 0.20 (95%CI:0.07 ~ 0.56), the difference was statistically significant. In the Caucasian population, the difference was not statistically significant. The heterogeneity decreased remarkably. The subgroup analysis of HWE showed that the decrease of heterogeneity was not obvious. The funnel plot was basically symmetrical (Fig. 3B), and Egger’s Test showed that *P* > 0.05, which indicated that there was no publication bias.

#### Recessive genetic model

In the recessive genetic model (CC vs. CT + TT), genotype CC was used as the exposure factor, genotype CT + TT as the non-exposure factor, and chemosensitivity as the control. The heterogeneity test showed that I^2^ = 28.7% (*P* > 0.05), indicating that the heterogeneity across studies was not significant. Therefore, a FEM was used. The forest plot was as shown in Fig. 2C. The results showed that OR = 0.85 (95%CI:0.69 ~ 1.05, *P* > 0.05), which was consistent with the result of the subgroup analysis of HWN. The ethnic subgroup analysis showed that in the Asian population, OR = 0.21 (95%CI: 0.07 ~ 0.59), the difference was statistically noteworthy. In the Caucasian population, the difference was not statistically meaningful. The subgroup analysis of HWE showed that there was no notable decrease in heterogeneity. The funnel plot was basically symmetrical (Fig. 3C), while Egger’s Test showed that *P* < 0.05, indicating a certain publication bias.

#### Homozygous genetic model

In the homozygous genetic model (CC vs. TT), genotype CC was used as the exposure factor, genotype TT as the non-exposure factor, and chemosensitivity as the control. The results of the heterogeneity test showed that I^2^ = 75.1% (*P* < 0.05), indicating that there were noteworthy differences in heterogeneity across studies. Hence, a REM was adopted. The forest plot was as shown in Fig. 2D. The results showed that OR = 0.62 (95% CI:0.26, 1.48, *P* > 0.05). The ethnic subgroup analysis showed that for the Asian population, OR = 0.21 (95%CI: 0.07 ~ 0.59), the difference was statistically significant. In the Caucasian population, the difference was not statistically meaningful. The heterogeneity decreased apparently. Subgroup analysis of HWE showed that the decline in heterogeneity was not significant. The funnel plot was basically symmetrical (Fig. 3D), and Egger’s Test showed that *P* > 0.05, indicating that there was no publication bias.

#### Heterozygous genetic model

In the heterozygous genetic model (CT vs. TT), genotype CT was set as the exposure factor and genotype TT as the non-exposure factor. The results of the heterogeneity test showed that I^2^ = 74.4% (*P* < 0.05), indicating that there was a statistically significant difference in heterogeneity between studies. Therefore a REM was adopted. The forest plot was as shown in Fig. 2E. The results showed that OR = 0.56(95%CI:0.25 ~ 1.27, *P* > 0.05). The ethnic subgroup analysis showed that for the Asian population, OR = 0.19 (95%CI:0.07 ~ 0.54), the difference was statistically meaningful. In the Caucasian population, the difference was not statistically noteworthy. The heterogeneity decreased substantially. While the subgroup analysis of HWE showed that the heterogeneity did not decrease. The funnel plot was basically symmetrical (Fig. 3E), and Egger’s Test showed that *P* > 0.05, which indicated that there no obvious publication bias.

### Sensitivity analysis

**Fig. 4:**
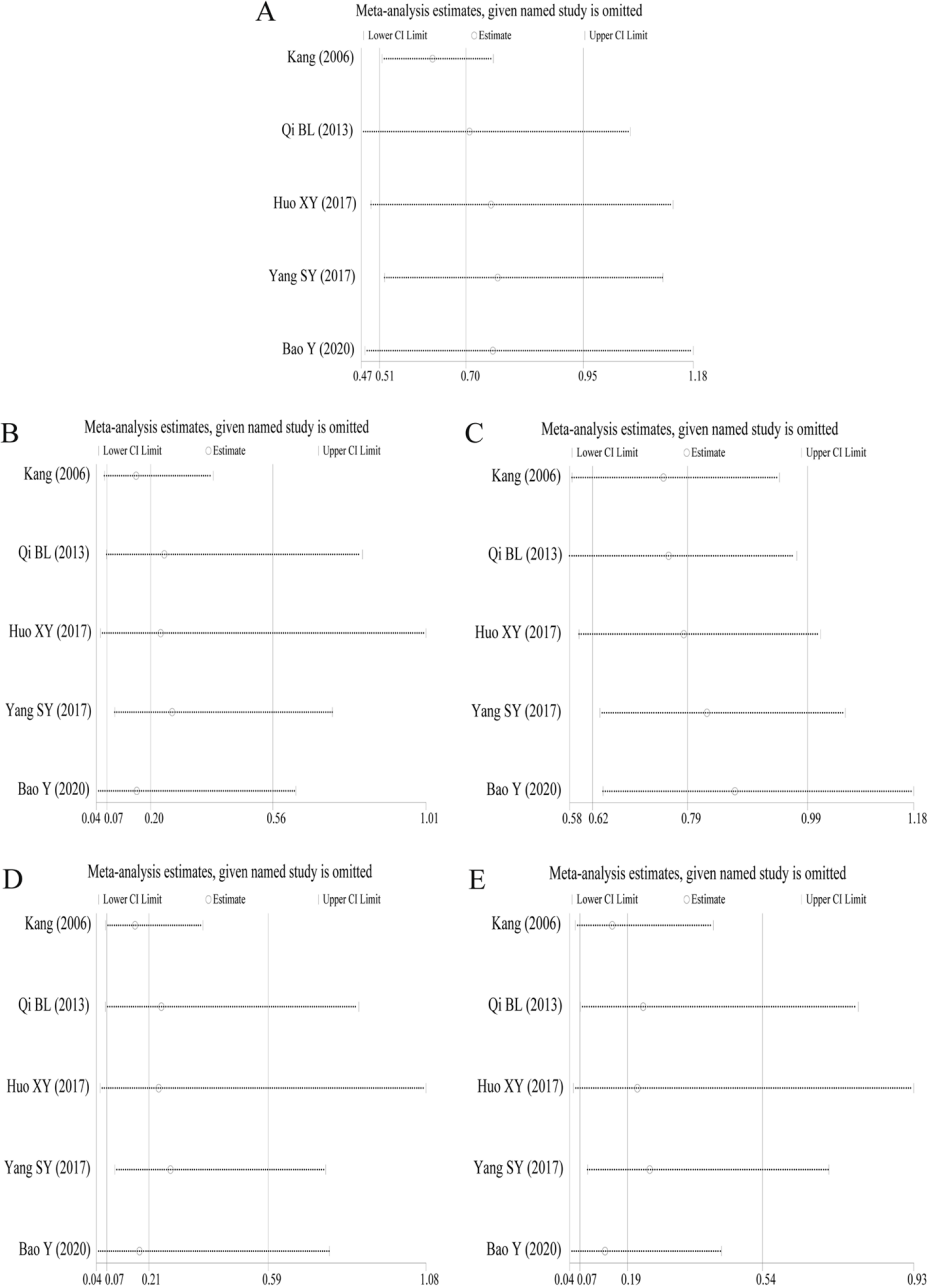
Results of sensitivity analysis of Asian population (**A** allele model; **B** dominant gene model; **C** recessive gene model; **D** homozygous model; **E** heterozygous model)

In consideration of the correlation between ERCC1 rs11615 polymorphism and platinum chemosensitivity of ovarian cancer in the Asian population, a targeted sensitivity analysis in the Asian population was conducted (Fig. 4A-E). The results showed that in the allelic, dominant, recessive, and homozygous genetic models, when the number 4, 1, 3, and 1 studies were eliminated, and the differences became insignificant. While in the heterozygous genetic model, with any study excluded, the conclusion did not change. As the above shows, there is a correlation between ERCC1 rs11615 polymorphism and platinum chemosensitivity of ovarian cancer in the Asian population. Meanwhile, allele C, genotype CC, and CT can increase the sensitivity of ovarian cancer patients in the Asian population to platinum chemotherapy. However, it still needs careful interpretation in allele, dominant, recessive, and homozygous genetic models.

## Discussion

At present, the conclusions upon the rs11615 polymorphism of the ERCC1 gene and the chemosensitivity of platinum drugs in ovarian cancer are not consistent. In order to explore the relationship between them, this meta-analysis was conducted, of which the results showed that there was a correlation between the rs11615 polymorphism of ERCC1 and the chemosensitivity of platinum drugs in ovarian cancer, mainly in the Asian population, but not in the Caucasian population. There are numerous factors that affect the sensitivity of chemotherapy, among which the polymorphism of ovarian oncogene and the chemosensitivity and clinical prognosis of platinum drugs have always been the focus of research [32, 33]. There are also plenty of studies on the relationship between ERCC1 gene polymorphism and platinum chemotherapy sensitivity in patients with ovarian cancer [21, 22, 24]. The ERCC1 gene is located on chromosome 9q13.2-q13.3, which is a DNA sequence containing 10 exons, with its length larger than 15 kb [34]. The gene encoding product is ERCC1 protein. In the NER pathway, ERCC1 protein and xeroderma pigmentosum complementation group D (XPD) together form a heterodimer of ERCC1-XPD, which is a 5ʹ-3ʹ DNA restricted endonuclease in NER, and also functions in DNA repair connection and inner chain cross repair [34]. These years, although studies have shown that overexpression of DNA repair genes can change the ability of DNA repair [35, 36], its regulatory mechanism is obscure yet. Some studies have suggested that single nucleotide polymorphism (SNP) can change the expression of repair genes [37–39], while other studies have shown that abnormally hypermethylation in the promoter region can inhibit gene expression, which will subsequently lead to the occurrence of some diseases and tumors and the resistance of chemotherapeutic drugs [40, 41]. ERCC1 gene is the dominant gene in NER and an important component of DNA damage identification and repair, of which the expression and single nucleotide polymorphism may affect platinum resistance and prognosis in patients with ovarian cancer [6, 42].

This meta-analysis eventually included ten studies with a total of 1866 patients with ovarian cancer. The results showed that the rs11615 polymorphism of the ERCC1 gene was strongly associated with ovarian cancer patients in the Asian population, and the differences were statistically important in allele, dominant, recessive, homozygous, and heterozygous genetic models. However, no such correlation was discovered in the Caucasian population. In accordance with the ethnicity subgroup analysis, whether in the Asian population or the Caucasian population, the heterogeneity declined significantly. A subgroup analysis was also conducted on whether the genotype frequency of the control group meets HWE, the results of which showed that according to the HWE subgroup analysis, the heterogeneity in each gene model did not notably decrease. The sensitivity analysis results in the Asian population showed that the conclusion in the heterozygous model was robust, but after the allelic, dominant, recessive, homozygous, and heterozygous genetic models were removed, the difference became not statistically significant. In each gene model, the funnel plot was approximately symmetrical. The *P-*value of Egger’s Test in both allele model and recessive gene inheritance model was less than 0.05, indicating that there was a certain publication bias in the two genetic models. As to other genetic models, the *P* values of Egger’s Test were all greater than 0.05, which suggested that there was no significant publication bias in these genetic models. Consequently, it can be concluded that the rs11615 polymorphism of the ERCC1 gene is associated with ovarian cancer patients in the Asian population. Allele C, genotype CC, and CT will increase the sensitivity of ovarian cancer patients in the Asian population to platinum chemotherapy, but some genetic models still need to be explained carefully. Tang et al. [43] reported that there is no correlation between the rs11615 polymorphism of the ERCC1 gene and the chemosensitivity of platinum drugs in ovarian cancer, which is inconsistent with our conclusion. This may be for the reason that more up-to-date and high-quality researches were included in this study. Li et al. [44] reported that the genotype TT at ERCC1 rs11615 locus increases the risk of death in patients with ovarian cancer after platinum chemotherapy, which is consistent with this study to some extent. A meta-analysis by Yang et al. in 2019 [45] showed that ERCC1 rs11615 polymorphism was not associated with chemosensitivity in patients with ovarian cancer, and a subgroup analysis of the Asian population showed the same conclusion. This is different from our conclusion. Our study showed that all genetic models were statistically significant in the Asian populations. Although only five studies were included in the analysis of Asian population, the sensitivity analysis confirmed the robustness of the conclusion in the heterozygote model. This has critical clinical significance, which not only points out a key direction for further research, but also provides a crucial basis for individualized treatment of ovarian cancer patients according to ERCC1 gene polymorphism in the future.

At present, our study found that there is a correlation between ERCC1 rs11615 polymorphism and chemotherapy sensitivity of ovarian cancer in the Asian population, but not in the Caucasian population. It may be that there are differences between chemotherapy sensitivity and ovarian cancer patients of various ethnicity. It has been reported that several differences exist in chemotherapy sensitivity to gynecological tumors in different races [46]. In addition, it is possible that the included studies are limited, resulting in no differences in the Caucasian population.

Inevitably, this study is limited by the following factors. First, the number of literature included was relatively small. After ethnicity subgroup analysis, there were only five studies in the Asian population and the Caucasian population, respectively, which had a certain impact on the robustness of the conclusion. Second, the sample size was relatively small, which was likely to affect the testing efficiency of statistics. Third, there was a certain publication bias in allele model and recessive gene model. Last but not least, in the sensitivity analysis of the Asian population, only the conclusion of the heterozygous genetic model was stable, while the conclusion of other genetic models was unstable to a certain degree.

In conclusion, the ERCC1 rs11615 polymorphism is associated with the sensitivity of platinum-based combination chemotherapy in patients with ovarian cancer, especially in the Asian population. Allele C, genotype CC, and CT will increase the sensitivity of ovarian cancer patients to platinum chemotherapy in the Asian population. Nevertheless, no such correlation has been found in the Caucasian population. Given that there are certain limitations in this study, such as small sample size and publication bias, the conclusions of this study still need to be further verified by larger sample size and high-quality clinical research.

## Data Availability

The datasets used and/or analysed during the current study are available from the corresponding author on reasonable request.

## Abbreviations

CNKI: China National Knowledge Infrastructure
NOS: Newcastle–Ottawa scale
REM: Random-effects model
FEM: Fixed-effects model
HWE: Hardy–Weinberg Equilibrium
OR: Odds ratio
NER: Nucleotide excision repair
XPD: Xeroderma pigmentosum complementation group D
SNP: Single nucleotide polymorphism
